# Footwear has a modifying effect on tibial loading during military weight carriage

**DOI:** 10.1038/s41598-025-11202-8

**Published:** 2025-07-22

**Authors:** Sanghyuk Han, Jongchul Park, Jusung Lee, Matthew Ellison, Dominic Farris, Hannah Rice

**Affiliations:** 1 Public Health and Sport Sciences, University of Exeter, Exeter, EX2 4TH UK; 2 Department of Marine Sports, Pukyong National University, Busan, 48513 Republic of Korea; 3 Human Performance Laboratory, Descente Innovation Studio Complex, Busan, 46772 Republic of Korea; 4 Department of Physical Performance, Norwegian School of Sport Sciences, Oslo, 0863 Norway

**Keywords:** Load carriage, Military boots, Walking, Bending moment, Cumulative loading, Tibial stress injury, Orthopaedics, Bone, Occupational health, Bone

## Abstract

This study investigated how different footwear conditions influence tibial loading across incremental load carriage during walking. Ten military-trained male participants completed walking trials under three weight conditions (0, 15, and 30 kg) and three footwear conditions (barefoot, trainers, and military boots) at 1.67 m/s. Kinematic (120 Hz) and kinetic (1200 Hz) data were collected using motion capture and force plates. Tibial loading was estimated via musculoskeletal modeling and beam theory, focusing on peak tibial bending moments and cumulative-weighted tibial impulse. A two-way repeated measures ANOVA ($p < 0.05$) examined main effects and interactions of load and footwear. Post hoc pairwise comparisons with Bonferroni corrections ($p_{\text {corr}} < 0.05$) identified significant differences. A significant interaction effect was observed for peak tibial bending moments and cumulative-weighted tibial impulse per kilometer ($p < 0.05$). In trainers, tibial loading increased progressively across all loads (0 kg < 15 kg < 30 kg, all $p_{\text {corr}} < 0.05$). In military boots, loading increased from 0 to 15 kg ($p_{\text {corr}} < 0.05$) but not between 15 and 30 kg. Weight carriage increased tibial loading, but footwear modified this relationship. Military boots showed no significant change between 15 and 30 kg. These findings suggest implications for tibial stress injury, though further research is needed.

## Introduction

Tibial stress injuries are a major concern among military personnel, particularly affecting recruits during basic training^1^. These injuries are understood to arise from repetitive mechanical stress and abrupt increases in physical activity, such as prolonged walking and running with heavy weights^2–4^. Stress fractures in the tibia account for 14–74% of all stress fractures in military populations, often leading to prolonged training interruptions and reduced operational readiness^3,5,6^. Given the high physical demands placed on military personnel, understanding the biomechanical factors that contribute to tibial stress injuries is essential for injury prevention and performance optimization.

Load carriage, referred to in this study as weight carriage in order to distinguish it from “tibial loading”, is an essential component of military operations, requiring personnel to transport heavy equipment over long distances. This additional weight, alters gait mechanics, increases mechanical stress on the lower extremities^7,8^ and has the potential to increase the risk of bone stress injuries. Previous studies have shown that increasing carried weight leads to increased ground reaction forces, increased braking forces during initial stance, and increased joint contact forces, all of which may contribute to musculoskeletal fatigue and stress-related injuries^9,10^. Recent research indicates that increased weight carriage during running elevates peak and cumulative tibial loading, potentially heightening the risk of tibial stress injuries^7^.

Footwear plays a critical role in modulating biomechanical responses to weight carriage. Military boots, in particular, differ from athletic footwear in key design characteristics, including higher shafts, greater mass and increased stiffness, which may enhance stability but also alter natural joint movement^11,12^. Previous studies suggest that military boots reduce ankle and knee range of motion, leading to compensatory adjustments at the hip and changes in overall force distribution^11,13^. Furthermore, while some military boots have lower shock absorption properties compared to athletic footwear, their increased stiffness and structural design may contribute to mechanical load redistribution^11,12^. Studies have shown that military boots influence gait mechanics by modifying stride length and altering joint range of motion, which may affect force transmission and lower limb kinematics in a manner distinct from athletic footwear^11,14^. Nevertheless, the extent to which these biomechanical adaptations influence tibial loading remains unclear, and thus the interaction between weight carriage and footwear during walking warrants further investigation.

Understanding internal tibial loading is essential for accurately assessing stress injury risk, as external forces such as ground reaction forces alone do not fully reflect the mechanical demands placed on the bone^15^. While invasive methods, such as strain gauges, have provided direct tibial loading measurements, recent research has shifted toward non-invasive approaches, including musculoskeletal modeling to estimate tibial loading magnitude^6,16,17^. Although the magnitude of loading is considered the primary factor that influences tibial stress accumulation, the number of loading cycles and the duration of loading also play a significant role^18,19^. These factors can be quantified using a weighted impulse method, which incorporates both magnitude and the quantity of exposure to loading provide a more comprehensive assessment of cumulative tibial loading^20^.

The purpose of this study was to evaluate the combined influences of incremental weight carriage (0 kg, 15 kg, and 30 kg) and different footwear conditions (barefoot, trainers, and military boots) on tibial loading during walking. Specifically, peak tibial bending moments and cumulative-weighted tibial impulse per kilometer were analyzed to assess tibial loading responses to varying weights and footwear types. Additionally, ankle joint mechanics and spatiotemporal gait parameters were examined to better understand the mechanical factors contributing to changes in tibial loading. It was hypothesized that tibial loading would increase with additional weight, but that the effect would differ across different footwear conditions.

## Results

### Tibial loading metrics

#### Tibial bending moments

**Fig. 1:**
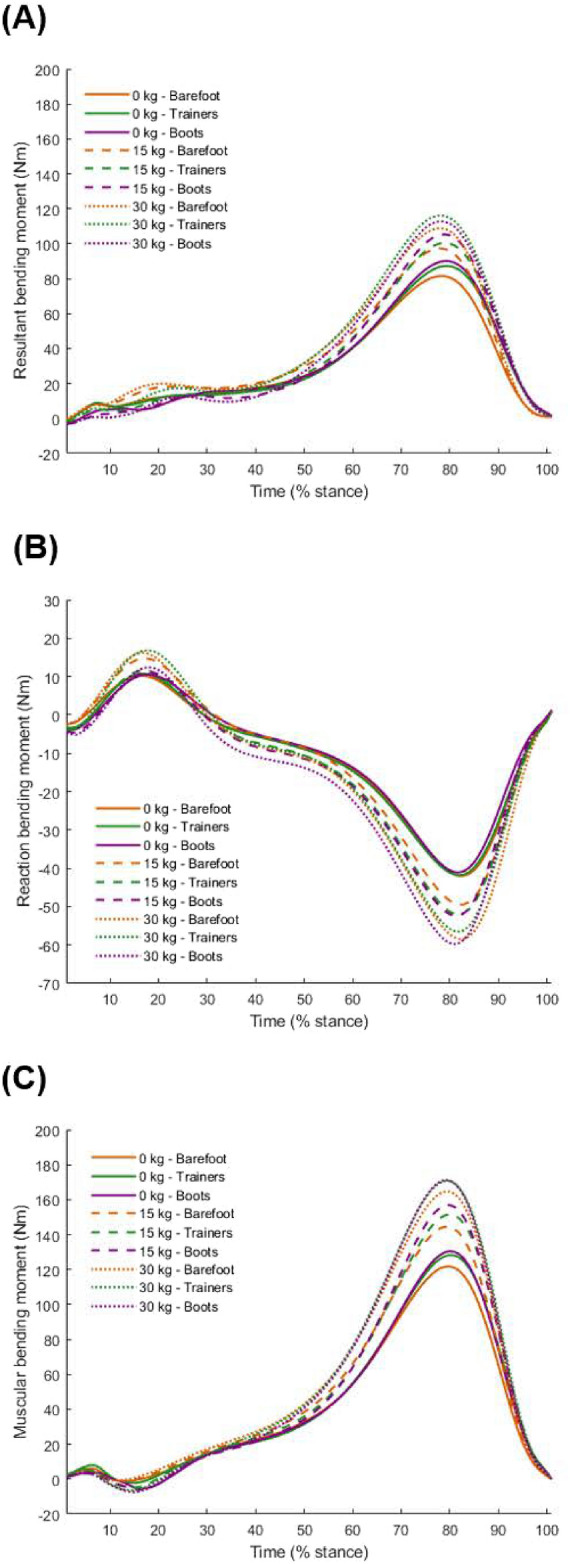
Mean time-series data of resultant bending moment (**A**), JRF component of bending moment (**B**), and muscular force component of bending moment (**C**) during the stance phase of walking under different weight conditions (0 kg, 15 kg, and 30 kg) and footwear conditions (barefoot, trainers, and military boots).

**Fig. 2:**
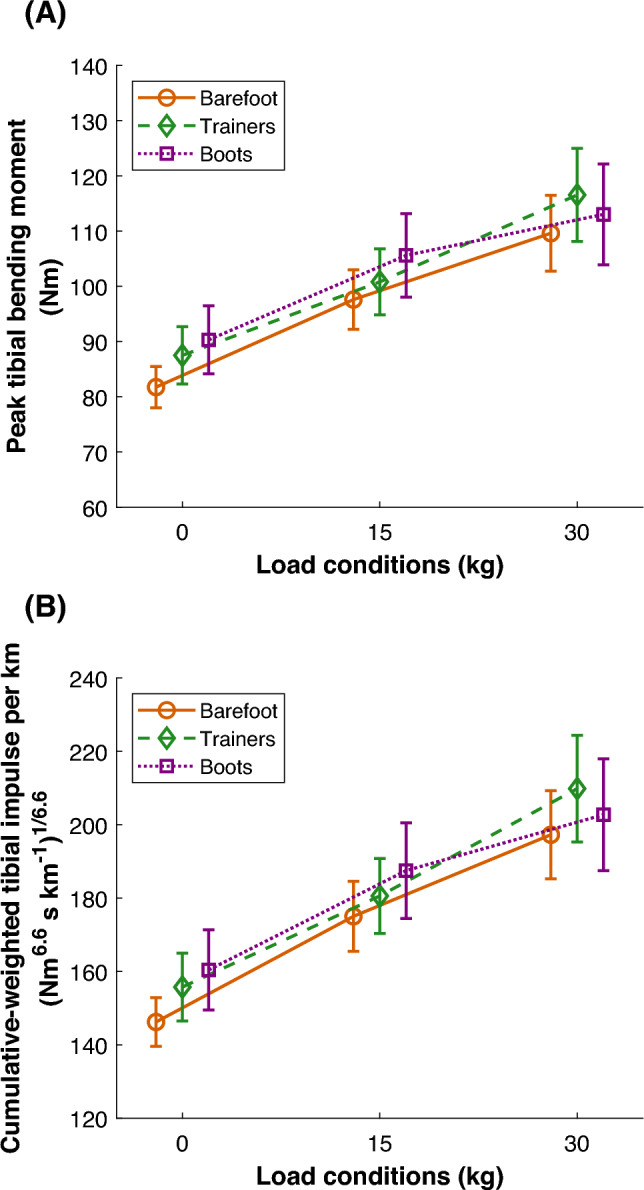
Mean peak tibial bending moment (**A**) and cumulative-weighted tibial impulse per kilometer (**B**) at the distal third of the tibia during the stance phase of walking under three weight conditions (0 kg, 15 kg, and 30 kg) and three footwear conditions (barefoot, trainers, and military boots). Error bars represent the standard deviation.

The tibial bending moments indicate compression posteriorly and tension anteriorly throughout stance (Fig. 1). A significant interaction effect was observed for peak resultant bending moments ($F_{(4,36)} = 3.133$, $p = 0.026$, Table 1). In trainers, peak resultant bending moments increased significantly with each incremental weight (0 kg < 15 kg, 0 kg < 30 kg, and 15 kg < 30 kg, all $p_{\text {corr}} < 0.05$) whereas in military boots, peak resultant bending moments increased significantly from 0 to 15 kg ($p_{\text {corr}} < 0.05$) and from 0 to 30 kg ($p_{\text {corr}} < 0.05$) (Fig. 2A). However, there was no significant difference between 15 and 30 kg ($p_{\text {corr}} = 0.324$) (Fig. 2A). In the barefoot condition, peak resultant bending moments also increased significantly with each incremental weight (all $p_{\text {corr}} < 0.05$). Furthermore, no statistically significant differences were observed between footwear conditions within the same weight conditions.

No interaction effect was found for the JRF Component of peak bending moment ($F_{(4,36)} = 1.566$, $p = 0.204$, Table 1). However, a significant main effect for weight conditions was observed ($F_{(1.125,10.123)} = 57.448$, $p < 0.001$), indicating that the magnitude of the JRF Component increased by 23.2% from 0 to 15 kg ($p_{\text {corr}} < 0.05$), by 13.7% from 15 to 30 kg ($p_{\text {corr}} < 0.05$), and by 40.0% from 0 to 30 kg ($p_{\text {corr}} < 0.05$). There was no main effect for footwear conditions ($F_{(2,18)} = 0.890$, $p = 0.428$).

No interaction effect was found on the Muscular Component of peak bending moment ($F_{(4,36)} = 1.854$, $p = 0.140$, Table 1). A significant main effect of weight conditions was observed ($F_{(1.178,10.605)} = 162.590$, $p < 0.001$), indicating that the Muscular Component increased by 19.0% from 0 to 15 kg ($p_{\text {corr}} < 0.05$), by 12.0% from 15 to 30 kg ($p_{\text {corr}} < 0.05$), and by 33.3% from 0 to 30 kg ($p_{\text {corr}} < 0.05$). However, while a main effect for footwear conditions was found ($F_{(2,18)} = 5.191$, $p = 0.017$), *post-hoc* pairwise comparisons revealed no significant differences between footwear conditions averaged over weight conditions.

**Table 1:** Mean (SD) tibial loading variables under three weight conditions (0 kg, 15 kg, and 30 kg) across three footwear conditions (barefoot, trainers, and military boots), along with results from two-way repeated measures ANOVA ($p < 0.05$).

Variable	Barefoot			Trainers			Military boots			W $\times$ F	Weight	Footwear
	0 kg	15 kg	30 kg	0 kg	15 kg	30 kg	0 kg	15 kg	30 kg			
Resultant bending moment (Nm)	81.7 (11.8)	97.6 (17.1)	109.6 (21.7)	87.5 (16.4)	100.8 (18.9)	116.5 (26.7)	90.3 (19.5)	105.6 (23.9)	113.0 (28.9)	***p*** = **0.026** $\eta ^2_p$ = 0.258	***p*** < **0.001** $\eta ^2_p$ = 0.832	*p* = 0.078 $\eta ^2_p$ = 0.247
JRF Component bending moment (Nm)	− 42.18 (5.84)	− 49.72 (7.00)	− 58.75 (9.23)	− 41.94 (3.68)	− 52.13 (6.40)	− 56.77 (9.49)	− 41.17 (5.33)	− 52.49 (6.99)	− 59.93 (7.56)	*p* = 0.204 $\eta ^2_p$ = 0.148	***p*** < **0.001** $\eta ^2_p$ = 0.865	*p* = 0.428 $\eta ^2_p$ = 0.090
Muscular Component bending moment (Nm)	122.0 (14.6)	144.9 (16.8)	165.4 (19.8)	128.5 (16.6)	151.7 (20.4)	171.2 (26.8)	130.7 (19.6)	157.1 (25.1)	171.5 (27.7)	*p* = 0.140 $\eta ^2_p$ = 0.171	***p*** < **0.001** $\eta ^2_p$ = 0.948	***p*** = **0.017** $\eta ^2_p$ = 0.366
Cumulative-weighted impulse ((Nm$$^{6.6}\cdot$$ s$$\cdot$$km$$^{-1})^{1/6.6}$$)	146.8 (20.9)	176.2 (30.3)	198.9 (38.0)	155.9 (29.0)	180.7 (32.2)	210.4 (45.0)	160.8 (34.8)	188.0 (41.2)	203.5 (48.8)	***p*** = **0.042** $$\eta ^2_p$$ = 0.235	***p*** < **0.001** $$\eta ^2_p$$ = 0.865	*p* = 0.118 $$\eta ^2_p$$ = 0.211

W $\times$ F indicates the interaction effect between weight and footwear.

#### Cumulative-weighted tibial impulse per kilometer

A significant interaction effect was observed for the cumulative-weighted tibial impulse per kilometer ($F_{(4,36)} = 2.759$, $p = 0.042$, Table 1). The cumulative-weighted tibial impulse per kilometer in trainers increased significantly with incremental weights (0 kg < 15 kg, 0 kg < 30 kg, and 15 kg < 30 kg, all $p_{\text {corr}} < 0.05$) (Fig. 2B). Whereas in military boots, the cumulative-weighted tibial impulse per kilometer increased significantly from 0 to 15 kg ($p_{\text {corr}} < 0.05$) and from 0 to 30 kg ($p_{\text {corr}} < 0.05$), no significant difference was observed between 15 and 30 kg ($p_{\text {corr}} = 0.090$) (Fig. 2B). In the barefoot condition, cumulative-weighted tibial impulse also increased significantly with each incremental weight (all $p_{\text {corr}} < 0.05$). Furthermore, no statistically significant differences were observed between footwear conditions within the same weight condition.

### Spatiotemporal variables

#### Ground contact time

No interaction effect was found for ground contact time ($F_{(4,36)} = 0.723$, $p = 0.582$, Table 2). However, a main effect for weight condition was observed ($F_{(1.171,10.536)} = 23.498$, $p < 0.001$), whereby ground contact time was 2.6% longer when carrying 15 kg than 0 kg ($p_{\text {corr}} < 0.05$), 2.4% longer when carrying 30 kg than 15 kg ($p_{\text {corr}} < 0.05$), and 5.0% longer when carrying 30 kg than 0 kg ($p_{\text {corr}} < 0.05$). Additionally, a significant main effect for footwear condition was found ($F_{(2,18)} = 28.348$, $p < 0.001$), where ground contact time was 5.0% longer in military boots than barefoot ($p_{\text {corr}} < 0.05$), 2.7% longer in military boots than trainers ($p_{\text {corr}} < 0.05$), and 2.1% longer in trainers than barefoot ($p_{\text {corr}} < 0.05$).

#### Stride frequency

No interaction effect was observed for stride frequency ($F_{(4,36)} = 0.718$, $p = 0.585$, Table 2). There was no main effect for weight condition ($F_{(1.140,10.262)} = 0.035$, $p = 0.884$). However, a significant main effect for footwear condition ($F_{(2,18)} = 16.717$, $p < 0.001$) was found. *Post-hoc* pairwise comparisons indicated that stride frequency was significantly lower in military boots than barefoot by 3.8% ($p_{\text {corr}} < 0.05$) and in trainers than barefoot by 2.8% ($p_{\text {corr}} < 0.05$), whereas the difference between military boots and trainers was not significant.

#### Stride length

No interaction effect was found for stride length ($F_{(4,36)} = 0.061$, $p = 0.993$, Table 2). There was no main effect for weight condition ($F_{(1.228,11.056)} = 1.835$, $p = 0.250$). However, a significant main effect for footwear condition was observed ($F_{(2,18)} = 48.060$, $p < 0.001$, $\eta ^2_p = 0.842$). *Post-hoc* pairwise comparisons showed that stride length was significantly longer in military boots than barefoot by 6.5% ($p_{\text {corr}} < 0.05$) and in trainers than barefoot by 5.8% ($p_{\text {corr}} < 0.05$), whereas the difference between military boots and trainers was not significant.

**Table 2:** Mean (SD) spatiotemporal variables and peak ankle joint plantarflexion moment under three weight conditions (0 kg, 15 kg, and 30 kg) across three footwear conditions (barefoot, trainers, and military boots), along with results from two-way repeated measures ANOVA ($p < 0.05$).

Variable	Barefoot			Trainers			Military boots			W $\times$ F	Weight	Footwear
	0 kg	15 kg	30 kg	0 kg	15 kg	30 kg	0 kg	15 kg	30 kg			
Ground contact time (ms)	604.06 (17.59)	625.13 (14.83)	638.17 (23.19)	626.10 (17.67)	637.64 (16.97)	652.11 (25.78)	635.41 (10.42)	652.42 (15.27)	669.67 (30.95)	*p* = 0.582 $\eta ^2_p$ = 0.074	***p*** < **0.001** $\eta ^2_p$ = 0.723	***p*** < **0.001** $\eta ^2_p$ = 0.759
Stride frequency (Hz)	1.04 (0.03)	1.03 (0.03)	1.03 (0.05)	1.00 (0.03)	1.01 (0.03)	1.00 (0.05)	0.99 (0.02)	0.99 (0.03)	0.99 (0.06)	*p* = 0.655 $\eta ^2_p$ = 0.064	*p* = 0.898 $\eta ^2_p$ = 0.012	***p*** < **0.001** $\eta ^2_p$ = 0.645
Stride length (m)	1.57 (0.10)	1.55 (0.09)	1.54 (0.09)	1.66 (0.12)	1.64 (0.11)	1.64 (0.12)	1.67 (0.11)	1.65 (0.08)	1.65 (0.09)	*p* = 0.993 $\eta ^2_p$ = 0.007	*p* = 0.188 $\eta ^2_p$ = 0.169	***p*** < **0.001** $\eta ^2_p$ = 0.842
Peak ankle joint plantarflexion moment (Nm/kg)	− 1.54 (0.11)	− 1.84 (0.13)	− 2.02 (0.22)	− 1.60 (0.10)	− 1.89 (0.13)	− 2.08 (0.21)	− 1.59 (0.16)	− 1.93 (0.18)	− 2.09 (0.25)	*p* = 0.534 $\eta ^2_p$ = 0.082	***p*** < **0.001** $\eta ^2_p$ = 0.905	*p* = 0.078 $\eta ^2_p$ = 0.247

W $\times$ F indicates the interaction effect between weight and footwear

### Sagittal ankle joint mechanics

#### Ankle plantarflexion moment

No interaction effect was found for peak ankle plantarflexion moment ($F_{(4,36)} = 0.799$, $p = 0.534$, Table 2). There was no main effect for footwear condition ($F_{(2,18)} = 2.957$, $p = 0.078$). However, a significant main effect of weight conditions was observed ($F_{(2,18)} = 85.405$, $p < 0.001$), indicating that the peak ankle plantarflexion moment increased by 19.8% as the weight increased from 0 to 15 kg ($p_{\text {corr}} < 0.05$), by an additional 9.3% from 15 to 30 kg ($p_{\text {corr}} < 0.05$), resulting in a total increase of 31.0% from 0 to 30 kg ($p_{\text {corr}} < 0.05$).

#### Ankle joint power

**Fig. 3:**
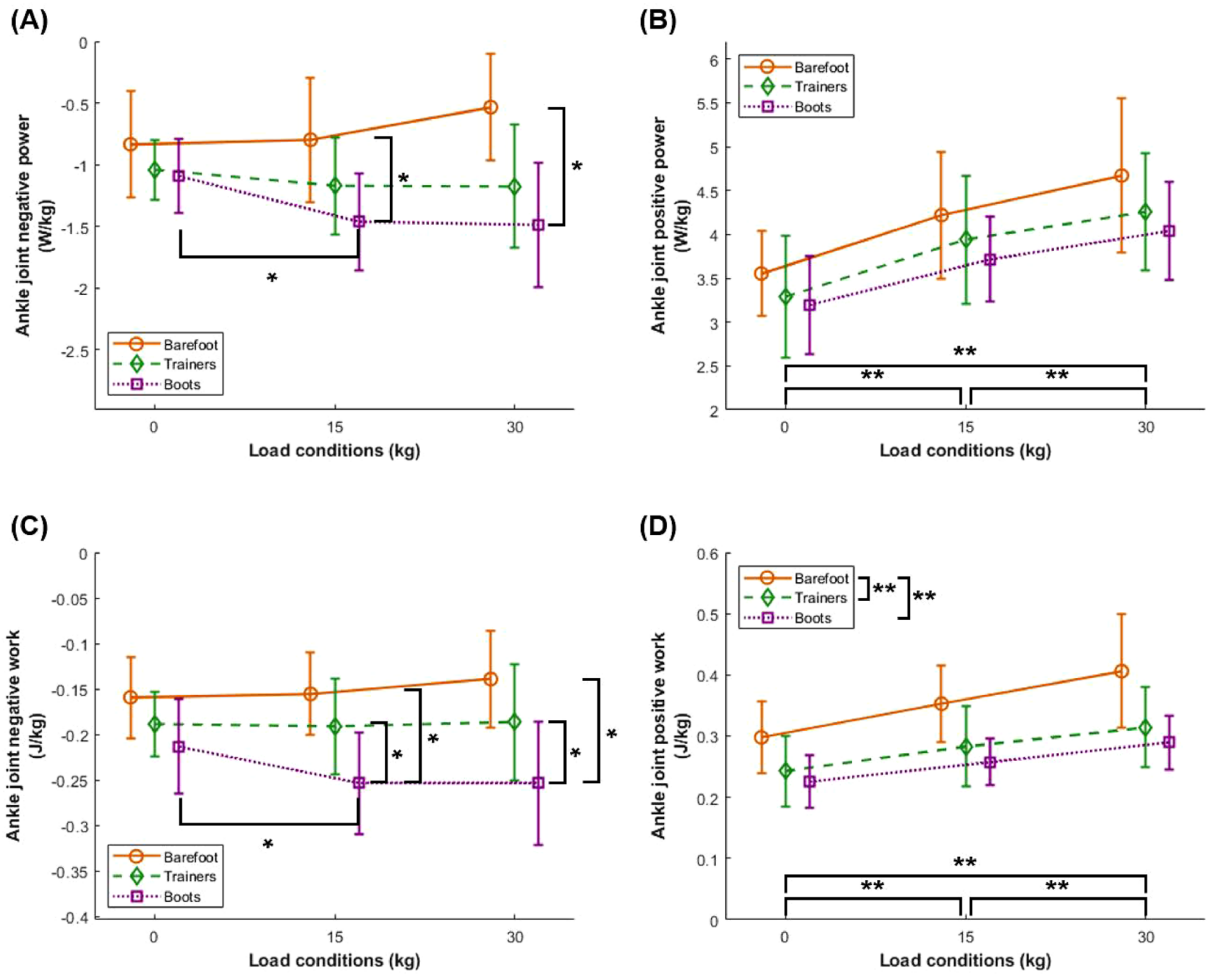
Ankle joint peak negative power (**A**), peak positive power (**B**), total negative work (**C**), and total positive work (**D**) across three weight conditions (0 kg, 15 kg, and 30 kg) and three footwear conditions (barefoot, trainers, and military boots). Error bars represent standard deviation. * indicates significant differences identified through *post-hoc* pairwise comparisons following a significant interaction effect, and ** indicates significant differences identified through *post-hoc* pairwise comparisons following a significant main effect, both at $p_{\text {corr}} < 0.05$.

A significant interaction effect was found for peak negative ankle joint power ($F_{(4,36)} = 10.621$, $p < 0.001$, Fig. 3A), indicating that the peak negative power differed significantly between barefoot and military boots when carrying 15 and 30 kg (barefoot < military boots, both $p_{\text {corr}} < 0.05$), but not at 0 kg. In military boots, peak negative power increased significantly from 0 to 15 kg ($p_{\text {corr}} < 0.05$).

There was no interaction effect on peak positive ankle joint power ($F_{(4,36)} = 0.607$, $p = 0.660$, Fig. 3B). A significant main effect of weight conditions was observed ($F_{(2,18)} = 40.174$, $p < 0.05$), whereby the peak positive power increased by 18.3% when weight increased from 0 to 15 kg ($p_{\text {corr}} < 0.05$), by an additional 9.2% from 15 to 30 kg ($p_{\text {corr}} < 0.05$), resulting in a total increase of 29.2% from 0 to 30 kg ($p_{\text {corr}} < 0.05$). However, while a main effect for footwear conditions was found ($F_{(2,18)} = 6.358$, $p = 0.008$), *post-hoc* pairwise comparisons revealed no significant differences between specific footwear conditions averaged across weight conditions.

#### Ankle joint work

A significant interaction effect was found for total negative ankle joint work ($F_{(4,36)} = 5.119$, $p = 0.002$, Fig. 3C). When carrying 15 and 30 kg, total negative work was significantly greater in military boots compared to both trainers and barefoot (both $p_{\text {corr}} < 0.05$). In military boots, total negative work also increased significantly from 0 to 15 kg ($p_{\text {corr}} < 0.05$).

There was no interaction effect on total positive ankle joint work ($F_{(4,36)} = 2.356$, $p = 0.072$, Fig. 3D). However, a significant main effect of weight condition was observed ($F_{(2,18)} = 45.624$, $p < 0.001$), indicating that total positive work increased by 16.9% from 0 to 15 kg ($p_{\text {corr}} < 0.05$), by an additional 13.1% from 15 to 30 kg ($p_{\text {corr}} < 0.05$), resulting in a total increase of 32.2% from 0 to 30 kg ($p_{\text {corr}} < 0.05$). A significant main effect of footwear was also observed ($F_{(2,18)} = 26.618$, $p < 0.001$). *Post-hoc* pairwise comparisons showed that total positive work was significantly lower in military boots than in barefoot by 26.7% ($p_{\text {corr}} < 0.05$) and in trainers than in barefoot by 20.5% ($p_{\text {corr}} < 0.05$), whereas the difference between military boots and trainers was not significant.

## Discussion

This study examined how different footwear conditions (barefoot, trainers, and military boots) and incremental weight carriage (0 kg, 15 kg, and 30 kg) influenced peak tibial bending moments and cumulative-weighted tibial impulse per kilometer during walking. The results demonstrated that while tibial loading generally increased with additional weight across footwear conditions, in military boots there was no significant difference between 15 and 30 kg, suggesting a potential role of military boots in load redistribution. This supports our hypothesis that tibial loading would increase with increasing weight but that footwear would have a modifying effect on this. This pattern was also observed in negative ankle joint power and work, indicating that military boots may influence how mechanical energy is absorbed and transferred through the lower limb under weight carriage conditions which may in turn influence or explain tibial loading mechanisms. The barefoot condition was included primarily as a baseline to mechanistically evaluate footwear effects. Pairwise comparisons across load conditions in the barefoot condition were conducted for completeness, though their interpretation remains limited due to lack of ecological relevance in military contexts.

Peak resultant tibial bending moments under the unloaded condition ranged from 81.7 Nm (barefoot) to 90.3 Nm (military boots), slightly exceeding the 70–80 Nm reported by Meardon et al.^21^ for sagittal tibial bending during unloaded walking at 1.3 m/s. The higher values observed in this study may be attributed to the faster walking speed (1.67 m/s). However, these values remain within a comparable range to previous studies, supporting the suitability of this study’s approach for quantifying tibial loading across different footwear and weight conditions.

There is limited research that has examined the effect of weight carriage on tibial loading across different footwear conditions during walking. The results of this study indicate a consistent increase in peak tibial bending moments with additional weight in all footwear conditions. This finding aligns with strain-gauge data reported by Yang et al.^22^, which showed that tibial bending increases in direct proportion to body mass. Similarly, computational models by Hughes et al.^23^ suggest that greater external weight contributes to elevated tibial strain. These results are further supported by Rice et al.^7^, who demonstrated that carrying an additional 20% of body weight increased peak tibial bending moment by 6.6% and cumulative-weighted tibial impulse by 8.5% during running. While these differences are lower than those observed in the present study, likely due to differences in locomotion (running vs walking) and load magnitude (20% body weight vs. fixed 15 and 30 kg weights), both highlight the impact of weight carriage on tibial loading. These findings reinforce the well-established principle that increased weight carriage results in greater tibial stress, emphasizing the importance of effective load distribution strategies in populations who are required to carry additional weight.

A key finding of this study was the significant interaction effect between weight and footwear conditions on ankle joint negative power and negative work (Fig. 3). Military boots exhibited greater negative work and therefore energy absorption than both barefoot and trainers when carrying weight. However, this greater energy absorption did not translate to greater energy return, which was lower in the military boot than barefoot, and not different between boots and trainers. This suggests a greater energy dissipation capacity at the ankle when wearing military boots than trainers or barefoot. While footwear stiffness can theoretically enhance energy storage and return, increased damping or altered joint mechanics may divert the absorbed energy into mechanical energy dissipation (hysteresis loss) rather than enabling elastic energy recycling. Böhm and Hösl^24^ demonstrated that greater shaft stiffness in military boots restricted ankle joint range of motion and redistributed energy absorption to the knee, consequently reducing concentric power generation at the ankle. Similarly, previous research indicates that stiffer footwear can alter ankle joint function, leading to increased energy absorption but diminished energy return during late stance^25,26^. These mechanisms may help to explain why peak tibial bending moments did not significantly increase between 15 and 30 kg of weight carriage in military boots, unlike in trainers. This suggestion in supported by the timing of peak positive ankle power which aligns closely with the time of peak tibial bending, likely due to the concentric contraction of the *triceps surae* muscle group. Consequently, instead of this energy being effectively utilized for propulsion, a greater proportion of energy may be dissipated under heavier loads in military boots, thereby minimizing the expected increase in tibial loading. With the carriage of even heavier weight, as occurs in applied military scenarios^27^, there may be a further attenuation of the increase in tibial loading compared with other footwear conditions, which could offer a protective effect. More studies are needed to determine long-term biomechanical adaptations and the possible protective effects of military boots under prolonged load carriage conditions.

To understand the interaction effect observed on the peak resultant tibial bending moment, the contributing components must be considered. The JRF and Muscular Components, which comprise the resultant bending moment act in opposite directions, where the Muscular Component has the greater magnitude. Therefore, although the JRF and the Muscular Components both increased with increasing weight, when wearing military boots, the JRF Component increased to a greater extent than the Muscular Component so that the resultant bending moment did not increase linearly as the weight increased from 15 to 30 kg. The structural characteristics of military boots, such as increased shaft support and material stiffness, may therefore have played a role in modulating load transfer in a manner that differs from trainers. However, in the military boot condition, markers were positioned on the boot rather than directly on the skin such that the boot is contributing to the ankle joint moments. As such, the findings represent the bending moments about the combined tibia and boot, rather than the tibia segment in isolation.

Interestingly, stride frequency decreased and stride length increased in military boots compared to barefoot and trainers, indicating an adjustment in walking mechanics. This aligns with previous findings comparing walking barefoot and in military boots^11,28^. These changes in spatiotemporal parameters may have influenced the observed tibial loading patterns. Although ground contact time was longer in military boots (653 ms) than trainers (639 ms, averaged across all weight conditions), cumulative-weighted tibial impulse per kilometer was not different between trainers and military boots with 30 kg weight, influenced in part by the lower stride frequency in boots but also by the non-significantly lower magnitude of the bending moments in boots than trainers. This interpretation aligns with previous research indicating that step frequency adjustments can modulate cumulative loading^29^.

Finally, long-term adaptation to military boots may influence lower limb loading patterns, potentially altering injury risk over time. While this study provides insight into acute biomechanical responses, further research incorporating muscle activity data and longitudinal gait adaptation analysis is needed to better understand how military boots influence tibial stress regulation during weight carriage conditions.

This study has several limitations that should be considered when interpreting the findings. Firstly, relying on computational modeling to estimate tibial loading presents inherent limitations. Although participant-specific anthropometric parameters were applied, the tibial geometry was simplified as a beam, which does not account for individual anatomical differences and does not consider material properties. Furthermore, the static optimization approach used in this study did not explicitly incorporate the structural influence of military boots on ankle joint kinetics. Military boots provide external support that may redistribute joint loading at the ankle and knee. Since the optimization model primarily considered muscle- generated forces without accounting for external support mechanisms, it may have led to an overestimation or underestimation of specific muscle activations and resultant joint moments. This is particularly important when considering that the ankle joint center was derived through markers positioned on the skin for the barefoot and trainer conditions but on the footwear for the military boot condition. This is often a challenge when assessing the influence of footwear conditions on foot and ankle mechanics using marker-based analyses. Although computational models provide valuable insights into internal loading, their inherent assumptions should be acknowledged when interpreting the results.

Secondly, the short-term nature of this study limits its ability to evaluate long-term biomechanical adaptations and cumulative injury risks associated with weight carriage. Military personnel are frequently exposed to prolonged weight carriage, which may lead to progressive musculoskeletal adaptations. However, this study only assessed acute biomechanical responses, such that the influence of fatigue and sustained exposure to footwear and weight carriage on tibial stress could not be assessed.

Additionally, the controlled laboratory environment and imposed footwear conditions may have influenced gait adaptations. Although participants were familiar with each type of footwear, the acute transitions between barefoot, trainers, and military boots may not fully reflect habitual use, potentially affecting gait mechanics. The short walking path in the laboratory setting means the gait characteristics may not truly replicate those typical in military operations or training. Furthermore, only one type of military boot was assessed in the present study, which is not representative of all styles of military boot.

Finally, the use of barbells placed inside a military backpack for weight simulation ensured precise weight control. However, this set-up does not represent the shape and weight distribution of actual military gear. Unlike real operational weight carriage systems, which involve asymmetric weights and shifting weight dynamics, the weight configuration had a fixed shape and center of gravity, potentially influencing body mechanics differently. Furthermore, participants did not wear standard military equipment such as belts or tactical vests, which are commonly used in the field and could further affect load distribution and posture.

## Conclusions

This study demonstrated that weight carriage during walking increases tibial loading, with footwear playing a role in modulating the mechanics of the lower extremities. Although the peak tibial bending moments increased progressively with additional weight in barefoot and trainers, the military boots exhibited a different pattern, with no significant difference observed between the 15 kg and 30 kg conditions. This suggests that military boots may facilitate load redistribution, potentially mitigating the increase in tibial loading that would be expected under heavier loads. These findings have implications for the risk of tibial stress injuries, highlighting the need for further research to evaluate long-term biomechanical adaptations to military footwear and the protective effects of load redistribution in prolonged operational scenarios.

## Methods

### Participants

Ten male participants (mean age: 24.1 ± 3.0 years; height: 1.77 ± 0.06 m; body mass: 76.7 ± 8.0 kg) were recruited for this study. All participants were physically fit with prior military experience, including heavy and prolonged weight carriage. Inclusion criteria required participants to be active duty soldiers or veterans with at least one year of military service and regular experience in military weight-carrying training. They also had to engage in physical activity at least three times a week. The exclusion criteria included any lower extremity musculoskeletal injuries in the past year. A sample size of ten participants was determined to be sufficient, based on previous studies with comparable load configurations and participant criteria^30,31^. All participants provided their informed written consent and the study was approved by the Institutional Review Board of the Korea Institute of Sport Science (IRB approval: KISS-1806-029-01). All methods were performed in accordance with the relevant guidelines and regulations.

### Experimental protocols

**Fig. 4:**
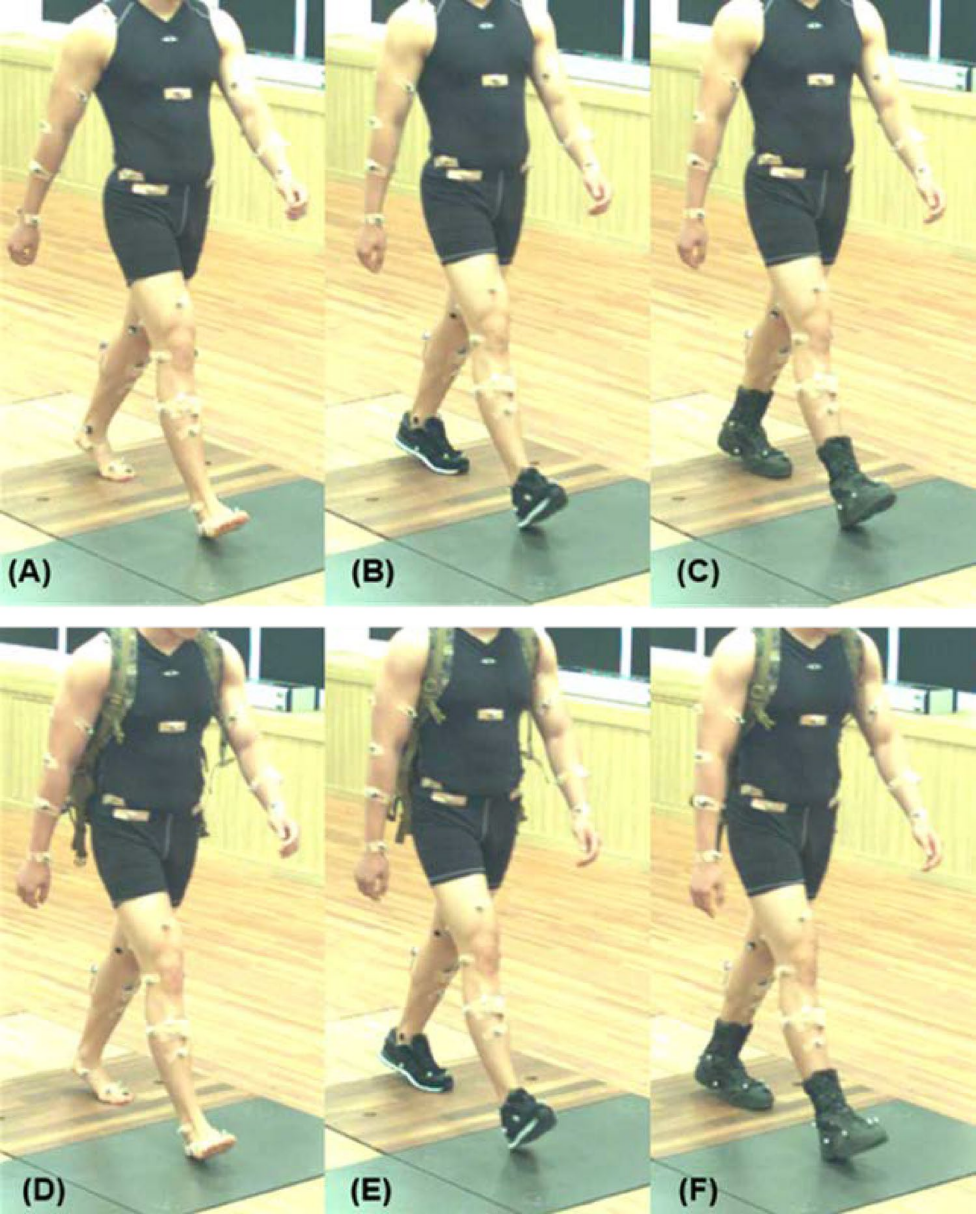
Examples of unloaded (0 kg) and loaded (15 and 30 kg) conditions during walking. (**A**) barefoot-unloaded; (**B**) trainers-unloaded; (**C**) military boots-unloaded; (**D**) barefoot-loaded; (**E**) trainers-loaded; (**F**) military boots-loaded.

The experiment was designed to compare tibial loading during walking under three different footwear conditions (barefoot, trainers and military boots) and three weight carriage conditions (0 kg, 15 kg, and 30 kg). Both trainers and military boots were compared with a barefoot condition, the latter not being relevant in a military setting but providing context for the magnitudes of difference observed between conditions. Load conditions of 15 and 30 kg were selected based on previous studies conducted with military personnel, in which 15 kg represented the weight of standard-issued body armor and equipment, while 30 kg included an additional assault pack to simulate heavier loads carried during military operations^10,32^. To achieve a consistent weight distribution, circular barbell plates were placed in a military backpack that was adjusted to ensure a stable and comfortable fit. A total of nine conditions (3 footwear x 3 weight combinations) were tested. The footwear conditions were presented in a random order, while the weight conditions were presented in increasing order (0 kg, followed by 15 kg, then 30 kg) (Fig. 4).

Fifty retro-reflective markers were placed to identify the anatomical frames of the thorax, pelvis, upper arm, forearm, thigh, shank and foot. Markers used to define the ankle joint center were placed directly on the skin over the medial and lateral malleoli for barefoot and trainer conditions. For military boots, markers were attached externally on the boot surface at the estimated malleoli locations. Marker coordinates were tracked at a sampling frequency of 120 Hz using an 18-camera motion capture system (Oqus 7+, Qualisys, Göteborg, Sweden). Ground reaction forces were recorded at a sampling frequency of 1200 Hz using a force plate (9287BA, Kistler, Winterthur, Switzerland).

Participants performed overground walking trials at a speed of 1.67 m/s, determined by prior weight carriage research^5^. Walking speed was monitored using two sets of timing gates (Witty, Microgate, BZ, Italy). A trial was considered successful if the right foot made full contact with the force plate and the walking speed remained within ±5% of the target speed. Each participant completed three successful trials for each weight condition (0 kg, 15 kg, and 30 kg). Trials were conducted in a controlled laboratory environment with consistent lighting and temperature conditions.

### Data processing

Marker position and ground reaction force data were processed using a low-pass, fourth-order, zero-lag Butterworth filter. For marker data, a cutoff frequency of 10 Hz was applied, while force data was filtered at 20 Hz. Kinematic and kinetic data were analyzed during the stance phase of walking using Visual3D software (V6; HAS-Motion, Ontario, Canada). The stance phase was defined as the time period during which the vertical component of the filtered ground reaction force was greater than 10 N. To enable comparison across all trials, the data were normalized to 101 time points, representing 0–100% of the stance phase. Stride length was determined using kinematic data by tracking the right heel marker, identifying foot contact as the first instance where its vertical displacement reaches a local minimum relative to the ground.

Static trials were conducted to determine the joint center locations for the ankle, knee, and hip on both sides for each participant. Joint angles were calculated using the XYZ Cardan sequence, where flexion-extension was represented on the X-axis, abduction-adduction on the Y-axis, and internal-external rotation on the Z-axis. Each joint was modeled with six degrees of freedom, accounting for three rotational and three translational movements. Net joint moments were calculated based on the right-hand rule, where counterclockwise rotation was assigned positive values. The joint power was determined by multiplying the net joint moments by the joint angular velocities, providing a measure of the rate at which mechanical energy was transferred or absorbed by the joint. Subsequently, the joint work was calculated by integrating the joint power over the stance phase to quantify the total energy absorbed (negative work) or generated (positive work) at the ankle. Joint reaction forces (JRF) were estimated using inverse dynamics in a customized MATLAB script (R2021a; MathWorks, Natick, MA, USA), which incorporated the anthropometric measurements of each participant, such as body mass, height, and sex^33^. These measurements were also used to derive segmental centers of mass and inertial properties.

To estimate muscular forces and tibial bending moments, custom MATLAB scripts were utilized, following previously established methods^17^. Muscle forces were estimated by static optimization using the fmincon function in MATLAB, which minimized the sum of cubed muscle stresses^34,35^. The optimization constrained muscle forces to produce joint moments consistent with experimentally measured sagittal-plane joint moments. This approach represented muscles as force vectors with constant moment arms and maximal isometric forces, and did not incorporate muscle activation dynamics or force-length-velocity relationships. The model included 11 muscles spanning the distal third of the tibia: lateral gastrocnemius, medial gastrocnemius, tibialis anterior, soleus, tibialis posterior, extensor digitorum longus, flexor digitorum longus, flexor hallucis longus, peroneus brevis, peroneus longus, and extensor hallucis longus. Muscle moment arms, as well as muscle-tendon coordinates including origins, insertions, and wrapping points, were based on data from the Hamner model^36^.

The resultant tibial bending moment ($M_{\text {resultant}}$) was estimated at 33% of the length of the tibia from the distal end, corresponding to the narrowest cross-sectional area of the tibia and a common site of stress fractures^37^. The robustness of the estimated tibial bending moments around the medial-lateral axis to variations in joint moment constraints has previously been evaluated^38^. Resultant bending moments were the sum of the muscular force component of the bending moment and the JRF component of the bending moment:

1 $$\begin{aligned} M_{\text {resultant}} = \left[ \sum _{i=1}^{11} F_{m_i} \cdot \sin \theta _i \cdot \left( L_{\text {tibia}} - L_{\text {67}\%\text {prox}} \right) \right] + \left[ F_{\text {JRF}} \cdot \sin \beta \cdot \left( L_{\text {tibia}} - L_{\text {67}\%\text {prox}} \right) \right] \end{aligned}$$

where $F_{m_i}$ represents the force generated by the $i$th muscle, $\theta _i$ is the sagittal plane angle between the tibial longitudinal axis and the force vector of the $i$th muscle, and $L_{\text {tibia}}$ denotes the total tibial length. $L_{\text {67}\%\text {prox}}$ is the distance from the proximal end of the tibia to the point of interest. Additionally, $F_{\text {JRF}}$ refers to the external JRF acting at the ankle, and $\beta$ is the sagittal plane angle between the tibial longitudinal axis and the resultant JRF vector. The muscular force component and the JRF components of the bending moment are referred to as the Muscular Component and the JRF Component, respectively. The Muscular Component included all modeled muscles spanning the distal third of the tibia. The cumulative-weighted tibial impulse was estimated to assess the mechanical loading of the tibia per kilometer of walking. This metric accounts for both the magnitude and duration of tibial bending moments during walking. The calculation follows the formulation proposed by Firminger et al.^20^ and incorporates per kilometer normalization as applied by Rice et al.^7^:

2 $$\begin{aligned} \text {Cumulative-weighted tibial impulse} = \left[ n \int _{t_i}^{t_f} \left( M_{\text {resultant}} \right) ^b dt \right] ^{\frac{1}{b}} \end{aligned}$$

where $n$ represents the number of right foot contacts per kilometer, calculated as:

3 $$\begin{aligned} n = \frac{1000 \text { m}}{\text {stride length (m)} \times 2} \end{aligned}$$

Stride length represents the distance covered in one full stride, defined as the distance between two consecutive right foot contacts. $t_i$ and $t_f$ denote the start and end times of the stance phase during walking, while $M_{\text {resultant}}$ is the internal tibial bending moment calculated using Eq. 1. The parameter $b$ was set to 6.6 for tibial bone, reflecting experimental findings that higher magnitudes of stress contribute disproportionately to the accumulation of bone damage^39^.

### Statistical analysis

A two-way repeated measures ANOVA was performed using IBM SPSS Statistics (Version 26; IBM, Chicago, IL, USA) to assess the main effects and interactions of weight carriage (0 kg, 15 kg, and 30 kg) and the conditions of footwear (barefoot, trainers and military boots) on internal tibial loading, spatiotemporal variables and sagittal plane ankle and knee joint kinetics. The assumption of sphericity was evaluated using Mauchly’s test, and where violations were detected, the Greenhouse-Geisser correction was applied. Effect sizes were measured using partial Eta$^2$ ($\eta ^2_p$) for the main effects, and interpreted as small (0.01–0.059), medium (0.06–0.139), and large ($> 0.14$)^40^. Statistical significance was set at $p < 0.05$.

In cases of significant interaction effects, *post hoc* analyses were conducted using paired *t*-tests between footwear conditions within each weight condition (3 footwear comparisons per weight condition), and between weight conditions within each footwear condition (3 weight comparisons per footwear condition). Thus, a total of 18 *post-hoc* comparisons were conducted. For variables where no interaction effect was found but a main effect was significant, *post hoc* analyses were conducted using paired *t*-tests with Bonferroni correction for 3 comparisons, either 3 weight comparisons across all footwear conditions or 3 footwear comparisons across all weight conditions. To account for multiple comparisons in these paired *t*-tests, Bonferroni-corrected *p*-values ($p_{\text {corr}}$) were calculated by multiplying the original *p*-values by the corresponding number of comparisons ($p_{\text {corr}} < 0.05$).

## Data Availability

The data that support the findings of this study are available from the corresponding author upon reasonable request.
